# The effectiveness of physical activity monitoring and distance counselling in an occupational health setting - a research protocol for a randomised controlled trial (CoAct)

**DOI:** 10.1186/1471-2458-9-494

**Published:** 2009-12-31

**Authors:** Karita Reijonsaari, Aki Vehtari, Willem van Mechelen, Timo Aro, Simo Taimela

**Affiliations:** 1 BIT Research Center, Helsinki University of Technology, Espoo, Finland; 2 Department of Biomedical Engineering and Computational Science, Helsinki University of Technology, Espoo, Finland; 3 Department of Public and Occupational Health, VU University Medical Center and EMGO+ Institute, Amsterdam, the Netherlands; 4 Mutual Pension Insurance Company Ilmarinen, Helsinki, Finland; 5 Department of Public Health, Helsinki University, Helsinki, Finland

## Abstract

**Background:**

The CoAct (Cocreating Activity) study is investigating a novel lifestyle intervention, aimed at the working population, with daily activity monitoring and distance counselling via telephone and secure web messages. The main purpose of this study is to evaluate the effectiveness of lifestyle counselling on the level of physical activity in an occupational health setting. The purposes include also analysing the potential effects of changes in physical activity on productivity at work and sickness absence, and healthcare costs. This article describes the design of the study and the participant flow until and including randomization.

**Methods/Design:**

CoAct is a randomised controlled trial with two arms: a control group and intervention group with daily activity monitoring and distance counselling. The intervention focuses on lifestyle modification and takes 12 months. The study population consists of volunteers from 1100 eligible employees of a Finnish insurance company. The primary outcomes of this study are change in physical activity measured in MET minutes per week, work productivity and sickness absence, and healthcare utilisation. Secondary outcomes include various physiological measures. Cost-effectiveness analysis will also be performed. The outcomes will be measured by questionnaires at baseline, after 6, 12, and 24 months, and sickness absence will be obtained from the employer's registers.

**Discussion:**

No trials are yet available that have evaluated the effectiveness of daily physical activity monitoring and distance counselling in an occupational health setting over a 12 month period and no data on cost-effectiveness of such intervention are available.

**Trial Registration:**

ClinicalTrials.gov identifier: NCT00994565

## Background

Lack of physical activity (PA) and exercise are known determinants and risk factors for many diseases and disorders, e.g., overweight and obesity, cardiovascular diseases, adult-onset diabetes mellitus and some types of cancer [1,2]. Globally more than one billion adults are overweight (i.e., having a Body Mass Index (BMI) > 25 kg/m2) already and the numbers are still rising [3]. For those who are physically inactive, becoming active is important to alleviate overweight related health problems and to reduce chances of developing cardiovascular diseases and diabetes [4]. PA and exercise may also alleviate the symptoms of distress, depressive mood [5] and sleeping problems, alleviate back pain [6] and thus contribute to productivity at work.

The work setting provides an opportunity to introduce a large group of adults to a lifestyle modification programme. Such programmes in health care settings usually rely on lifestyle modification to change dietary intake and/or physical activity [7]. These strategies are known to produce weight loss [8,9]. However, only two previous randomised trial have been published on the effectiveness of PA interventions in the occupational setting, and the results have not been encouraging [10,11].

Typically lifestyle modification is provided by (individual or group) face-to-face counselling, requiring multiple visits to a treatment facility. This may be less appealing to working adults, who are often constrained by lack of time for such programmes. Behaviour counselling over the Internet (i.e., distance counselling) could be more feasible in the work setting. In other settings distance counselling has been applied to weight loss, dietary behaviours and physical activity. Phone counselling trials for weight loss, including trials primarily aimed at changes in diet and/or physical activity, showed mixed results [12-15]. The majority of phone counselling studies for physical activity and dietary behaviour found behavioural changes [16]. Few trials have investigated e-mail or internet counselling for weight control or lifestyle behaviours. Those that did, found positive effects on body weight, mixed effects on diet [17,18] and no effect on PA [17-19]. A recent randomized controlled trial concluded that distance counselling by phone or email is effective for reducing body weight in a group of overweight employees [20]. Studies that recruit participants from the work setting have, however, been rare [17,20].

The physical activity monitoring and distance counselling concept combines the use of a personal activity monitor with web-based tailored physical activity advice. Users can interactively plan and evaluate their own activity advice based on their actual PA scores and their PA preferences and goals. A recent randomised controlled trial [21] evaluated the effectiveness of such online physical activity advice based on a personal activity monitor and found that a 3-month intervention did not significantly affect PA levels and, furthermore, that a higher adherence to the program did not result in increased levels of PA. The study group comprised of a total of 102 physically inactive employees, 23 to 39 years old.

The main purpose of the present study is to evaluate the effectiveness on physical activity of a lifestyle programme with daily monitoring of activity and distance counselling compared to self help instruction, in a large cohort with a broad age spectrum of employees, at 12 months. Employees in the intervention group are given accelometers to wear daily and they will receive counselling via telephone and secure web messages. Subsequent purposes are to determine effects of the distance counselling on work productivity, sickness absence, healthcare utilisation and health outcomes when comparing to self help.

In the study we will be analyzing the lifestyle intervention's influence to health behaviour and if changes in health behaviour affect overall health. We are also interested in the lifestyle program's effects on productivity at work, including the potential effects on sickness absence. Furthermore, we will analyse potential effect modifying and mediating factors.

### Eligible population

**Figure 1:**
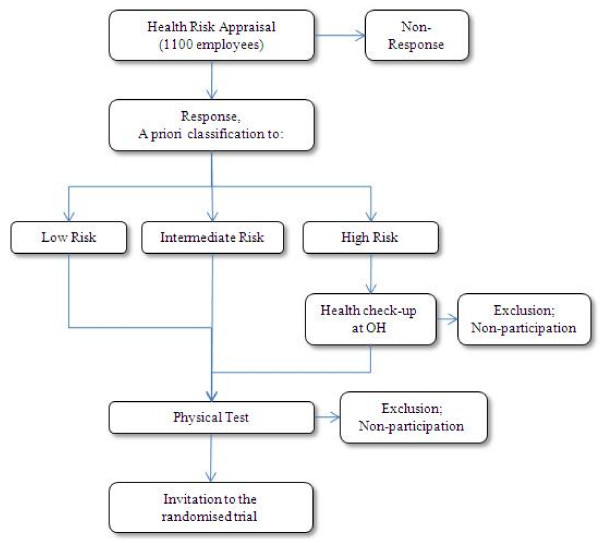
**Occupational healthcare procedures taking place before the intervention study**.

Participants are employees of a large insurance company in Finland. Before the study is started all employees are invited to respond a health risk appraisal as part of their normal occupational healthcare (Figure 1). All employees will be approached through an invitational email and they will receive a screening questionnaire. These emails will be sent out all at once during mid August 2009. Out of a total of approximately 1100 employees 75% are expected to return the baseline questionnaire. The 75 percent response rate is expected because of an information campaign and since eligible participants can be individually reminded to participate.

A self-administered questionnaire is sent to all employees. The questionnaire [22] contains previously validated items about anthropometrics, sleep disturbances, work-related stress and fatigue, depression, pain, disability due to musculoskeletal problems and a prediction of future work ability (Table 1).

**Table 1:** Items on the questionnaire and cut-off criteria for health problem

Topic	Questions	Criteria
Obesity	Height and weight, calculation of body mass index (BMI)	BMI ≥ 30
Alcohol consumption	Frequency and dosage. Modified from [36]	Alcohol/women: > 239 ml/abs alcohol/week: Alcohol/men: >349 ml/abs alcohol/week
Diabetes risk	Sum score of diabetes risk factors, scale 0-21; modification of DEHKO score [37]	Sum score ≥ 11
Pain	Frequency and intensity	At least "moderate" pain that "affects working ability" at minimum three times a week
Impairment due to musculoskeletal problems at work	Numerical rating scale (0-10) [38]	Impairment at work > 5
Depressive symptoms	Depression score DEPS, scale 0-30 [39]	DEPS ≥ 11
Stress and fatigue	Work related stress and fatigue [38,40]	"Very much" feeling of stress or fatigue because of work
Sleep disturbances	Modification of the Basic Nordic Sleep Questionnaire [41]	Problems in falling asleep or night awakenings AND daytime tiredness daily or almost daily
Daytime sleepiness	Epworth Sleepiness Scale, 0-24 [42]	ESS Score ≥ 11
Future working ability	Self-rated ability to continue working in the present job due to health problems after two years [43]	Uncertain of own ability ("Uncertain"), or quite sure ("Not able") not being able

The responses are interpreted on the basis of priori defined cut-off limits. Subjects who will report problems with future working ability, pain, impairment due to musculoskeletal problems, insomnia or insufficient sleep, frequent stress or fatigue, or have a high depression score, obesity, excess use of alcohol, or a high score in diabetes risk will be rated as having health problems (Table 1).

Based on self-reported health problems the employees are assigned to three groups: low-risk, intermediate and high-risk group. "High-risk" group is directed to a visit with occupational health. Employees receive advice or treatment and necessary follow up for their respective health problems. Targeted interventions at occupational health care for the "high risk" group have been shown to be effective in controlling sickness absence [23] and cost effective use of healthcare resources [24].

After the authorization of occupational physician the employee is directed to physical testing. "Low risk" and "intermediate risk" groups are scheduled for physical testing directly after receiving responses from the health risk appraisal. With the individual results employees are invited to schedule a time for physical testing through a web link. Most of the physical tests are done at the worksite during normal office hours. The physical test is done for voluntary participants as part of normal occupational healthcare.

### Physical testing

A background questionnaire concerning medical history and medication is filled in before physical testing. Employees can be excluded before or during the physical testing for multiple medical reasons: pregnancy, diagnosis or treatment of cancer, any disorder that makes physical activity impossible. Complete list of exclusion criteria is listed in Appendix 1. The test is started by measuring one's height, weight, waist circumference, body fat percentage and blood pressure. All measures are taken with previously validated measures and are described in more detail later. Aerobic fitness is measured by means of the indirect bicycle ergometer test (Aino Fitware). The test gives a prediction of the maximal oxygen uptake (VO2 max) during maximal exertion. The employee is asked not to eat, drink or smoke for 2 hours before this test. During the bicycle ergometer test the subject must report his/her subjective rate of exertion at the end of each stage using a scale of 6 to 20 [25]. The test is terminated when the pedalling rate drops under 60 rounds per minute. The estimated VO2 max is calculated with software analysis program (Aino Fitware). The AA Fitware test uses common validated procedures and measures from previous studies [26].

## Methods/Design

Once employees have completed the physical testing they will receive an invitation to participate in the randomised controlled trial. They will be handed an information leaflet in which the study protocol and the intervention options are clearly described. Employees can freely ask further about the study without any engagement. All employees willing to participate will individually sign an informed consent form that also allows for their personal data from the other data registers (health risk appraisal; physical testing; occupational healthcare utilisation; sickness absence records) to be collected to the research database and be used for the purposes of this study. The study received approval from the ethics review board (Helsinki University Hospital; Coordinating Ethics Committee) in August 2009.

CoAct (Coproducing Activity) is a randomized controlled trial with a two-year follow up. Randomization takes place at an individual level to two groups: to a control group and to an intervention group that receives a lifestyle intervention focusing on physical activity. The recruitment and data collection for this study will be started in August 2009. Data collection will continue until October 2011.

### Inclusion and exclusion criteria

Inclusion criteria of the study are: 1) Age 18 years or older, 2) paid employment of at least 8 hours a week 3) not scheduled to retire in the next two years or have applied for disability pension and 4) have completed the health risk appraisal and physical testing as part of normal occupational healthcare (picture 1). All subjects have an access to Internet and are skilled in using it.

### Randomization and blinding

Randomization will take place at an individual level. A biostatistician will prepare the randomization scheme by using a computer-generated randomization table. Block randomisation with blocks of ten will be applied. Based on the randomisation scheme, two research assistants will prepare sealed envelopes before the start of the study containing either a referral to the intervention group or control group. After signing the informed consent form, each employee will open a sealed envelope in the given order. The researchers will not be able to identify the group assignments before the randomisation. Due to the nature of the interventions, the participants and researchers will not be blinded for the group assignment after randomization. Employees will not be allowed to change groups after randomization. Sickness absence data will be extracted from the company records in an electronic format and self-reported data will be entered into the computer by a research assistant, which will ensure blinded analysis of the data by the researchers.

### Participant flow

**Figure 2:**
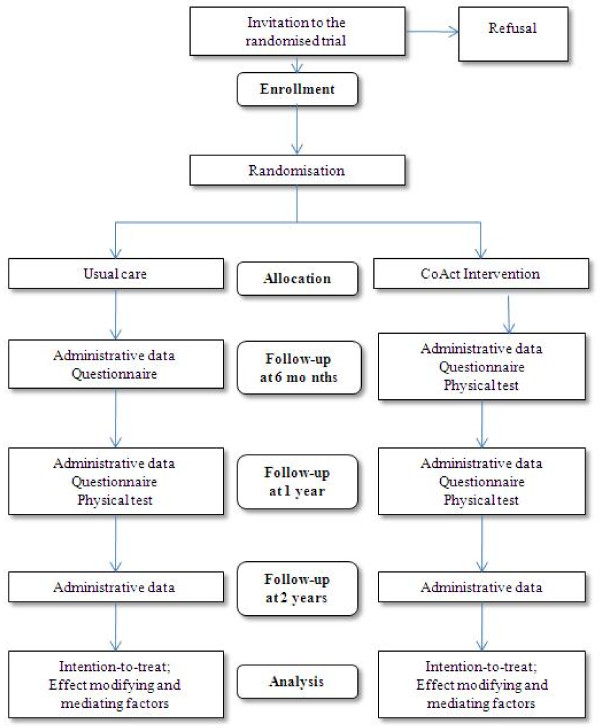
**Research design**.

The study design and participant flow are shown in Figure 2.

#### Control group

Employees in the control group will receive the results of the physical test in writing with general information on physical activity and health. The results and information material is briefly explained to the employee. Their occupational health care is continued as usual.

#### Intervention group

After the physical testing employees assigned to the intervention group will receive their test results and information on physical activity. They will also receive a physical activity monitor (PAM, model AM 200, PAM BV, the Netherlands), which is an uni-axial accelerometer in the vertical direction that can be easily attached to a belt. The validity of the PAM accelerometer has been tested in a laboratory setting and has shown results similar to the MTI actigraph for estimating energy expenditure in walking and stair walking [27]. The PAM produces a single index score that accumulates during the day and is a proxy measure of total daily PA. The PAM shows the PAM score continuously on its display.

The participants have to install the PAM software on their computer in order to use the PAM reader. Via a USB cable, which must be connected to a computer with an internet connection, the user can upload his or her personal PAM scores through PAM software to the Aino Active (AA) website http://www.ainoactive.fi at any time throughout the day. When reading the PAM, the participant is automatically directed to the AA website. During the first visit, the user will register on the AA website by filling out a form with personal data (ie, username and password). Upon entry to the website, the user formulates a PAM goal score for the 12 month intervention period. Based on the first two weeks the employee and AA's coach can together decide to change the PAM goal. The PAM goal score can be changed by the user and AA's coach throughout the intervention. On every subsequent log-in, the AA's website presents all the uploaded PAM scores and coupled PAM goals in orderly graphs per week or month.

On the Aino Active website, users can interactively plan and evaluate their own activity advice based on their actual PAM scores and their PA goals and preferences. When an employee does not log on to the site every two weeks to download activity data she/he receives a phone call from the coach. The subjects in the intervention group will also participate in a physical test at 6 months of the intervention. Their occupational health care is continued as usual.

### Outcome measures, confounding- and mediating variables

Primary outcomes of the study are 1) physical activity level measured in MET hours per week, 2) health parameters (musculoskeletal and depressive symptoms) 3) work productivity and number of sickness absence days 4) healthcare utilisation. Effectiveness of the intervention is assessed by comparing the outcomes between the two treatment arms. Secondary outcomes are also listed in the Table 2.

**Table 2:** Primary and Secondary outcome parameters

Outcome measure	Q, M, R	Baseline	6 months	12 months	24 months
**PRIMARY OUTCOMES**					
PHYSICAL ACTIVITY					
Physical Activity	Q	v	v	v	v
HEALTH RELATED					
Musculoskeletal disorders	Q	v		v	v
Depression score	Q	v		v	v
Healthcare service utilization	Q and R	v	v	v	v
PRODUCTIVITY PARAMETERS					
Sickness absence	R				
Work productivity	Q	v	v	v	v
**SECONDARY OUTCOMES**					
Body weight	M	v	v	v	
Body height	M	v			
Waist circumference	M	v	v	v	
Body fat percentage	M	v	v	v	
Blood pressure	M	v	v	v	
Aerobic fitness; VO2 Max	M	v	v	v	
Programme costs	R				

Q = Questionnaire, M = Measurement, R = Registry

Assessment of the secondary outcomes is done either by questionnaire, measurements during the physical test, or by collecting data from occupational healthcare provider and employer's absenteeism/payment records.

### Primary outcome measurements

#### Physical activity

The volume (frequency, intensity, duration) of leisure time and way to work physical activity are assessed by a self-administered questionnaire based on International Physical Activity Questionnaire (IPAQ). Total MET (metabolic equivalent) score MET min-per-week (continuous score from the IPAQ scoring protocol) is calculated as follows: (daily minutes of walking × days per week with walking × 3,3) + (daily minutes of moderate-intensity activity × days per week with moderate intensity activity × 4,0) + (daily minutes of vigorous activity × days per week with vigorous activity *8,0)[28] The MET values are derived from the IPAQ validity and reliability study [29]. In addition we will calculate truncated MET-minutes per week, in which all daily minutes exceeding 120 min will be truncated to 120 min. This rule has been proposed in the "Guidelines of Data Processing and Analysis of IPAQ Short Version" with the attempt to normalize skewed population data.

All participants will receive the physical activity questions as part of the questionnaire sent to them at the beginning of this study, as well as at 6 and 12 months from the intervention.

#### Work productivity

Work productivity is measured with the QQ instrument [30]. Respondents will be asked to indicate how much work they actually performed during regular hours on their last regular workday as compared with normal. The quantity of productivity is measured on 10-point numerical rating scale; 0 representing "nothing" and 10 representing "normal quantity" [30,31]. The self-reported productivity of the QQ instrument has been shown to correlate with the objective work output [31]. The questionnaire is sent to each employee at baseline, 6 and 12 months.

#### Sickness absence

Sickness absence days are analyzed from company's absenteeism and payroll records - each employee is required to inform the company when sick for one day to three days and required to get a certificate from occupational health when sick for longer than three days. Sickness absence data will also be collected for each employee from the year preceding the intervention in order to compare change over time.

#### Healthcare utilization

As part of the first questionnaire the employees will be requested to complete a questionnaire concerning healthcare resource use during the past 6 months. This questionnaire includes questions of different healthcare resources used. This questionnaire is presented again at the 6 and 12 month time points.

Healthcare utilization data is also retrieved from occupational healthcare records. Data is retrieved based on social security number and study period data is compared to data from preceding year on individual level.

### Secondary outcome measures

Secondary outcome measures are listed in Table 2 and are presented here in more detail.

#### Body Weight and body height

Body weight is measured in kg, to the nearest 0,1 kg with a digital scale (BC-418, MA). Participants are wearing light clothing and no shoes. Body height is measured in m, to the nearest 0.001 m with a portable meter (Charter HM 200P). Positioning of the body is standardized by asking the subject to stand straight, without shoes and heels together. Both weight and height are measured twice and for each mean value of the two measurements is computed.

#### BMI

BMI is calculated by dividing the measured body weight (kg) by the squared measured body height (m).

#### Waist circumference

Waist circumference (in cm) is measured twice with a measuring tape (Reiju Oy) with a range of 0-150 cm. Waist circumference is measured to the nearest 0,5 cm, at the midpoint between lower border of the ribs and the upper border of the pelvis. A means value of the two measurements is computed.

#### Body fat percentage

Body fat percentage is measured with a body fat analyzer device (TANITA) [32].

#### Blood pressure

Blood pressure is measured with a fully automated blood pressure monitor (Omron M3 Intellisense). A regular size cuff is used on the right upper arm. The right arm is placed on a table so as the cuff is on a level with the heart. After the employee has rested for 5 min in a sitting position, blood pressure is measured twice. A mean value of the two measurements is computed. Employees that have a blood pressure of 140/90 mmHg or higher are advised to visit their occupational healthcare provider.

#### Aerobic fitness

Aerobic fitness is measured by means of the estimated VO2 max during the indirect bicycle ergometer test.

### Sample size

The standard deviation for the IPAQ score in our population was estimated to be 1500 MET min-per-week. A difference of 400 MET min-per-week between treatment arms will be detectable with 85% power in two-tailed tests with the alpha of 0.05 for a sample of 253 employees in each group; the standardized effect size will be 0.25.

### Statistical analyses

The effect of the intervention will be estimated on an individual level based on the intention-to-treat principle. For each primary and secondary outcome, a summary of results for each group, and the estimated effect size and its precision with confidence intervals will be presented. We will also assess potential effect modifying and mediating factors. The potential modifiers and mediators have been predefined and will all be measured at baseline. The potential effect modifiers are personal characteristics (age and gender), the result of the health risk appraisal (high/intermediate/low risk), baseline self-rated level of physical activity, baseline level of the ergometer test; job characteristics (specialist/manager), sick-leave related characteristics (sick leave days in the past year), and self-rated disability thoughts. The potential effect mediating factors are attendance to the PA intervention as intended, healthcare utilisation, and sick-leave related characteristics (sick leave days during the follow-up year).

#### Cost-effectiveness analysis

Cost-effectiveness analyses (CEA) will be conducted from the societal perspective and cost-benefit analyses (CBA) from the employer perspective. To estimate cost and effect differences, relating confidence intervals and cost-effectiveness ratios bootstrapping techniques will be used. Cost-effectiveness planes and acceptability curves will be presented. Additionally, subgroup analyses will be performed. The cost of intervention includes costs that are directly related to the implementation of the program. Besides the cost of intervention itself, direct health care costs will be included. Also indirect costs resulting from a loss of production due to absenteeism from paid work will be analysed.

## Discussion

Decreased productivity at work is an important consequence of the presence of health problems. On the other hand numerous health problems can be prevented and treated with the right amount of daily physical activity. CoAct is motivated by the increasing interest in prevention in occupational health. The research presented here analyzes a lifestyle intervention's effects to health behaviour (changes in physical activity). Further on we will look at health behaviour effects on overall health, absenteeism and efficiency at work. The employee is considered a core part of preventive healthcare services as he or she can be in an important role as value cocreator, and contribute input to the healthcare process, such as enhanced levels of (daily) physical activity to avoid chronic conditions and to improve overall healthcare outcomes.

Some limitations of this study can be mentioned. Neither the self-administered questionnaires nor the accelometers are the perfect gold standard measure of physical activity [33-35]. Accelometers alone cannot provide contextual information about the type or purpose of specific activities. Also, due to the study design we can only track the change in physical activity for the whole population by the means of questionnaire that may be rather insensitive as an outcome measure.

Studying the effects of this intervention is important, as it contributes to our understanding on the effectiveness of this novel method of lifestyle intervention, physical activity monitoring with distance counselling. The intervention has in Finland been taken into clinical use, but randomised trials of the effectiveness of the method are yet almost lacking. This type of intervention has not been evaluated in this specific setting with such a significant population yet. We designed our study as pragmatic as possible for the purpose of studying the role of PA monitoring and distance counselling in the co-creation of value, namely physical activity. If proven effective the employee will benefit from an improved lifestyle and a reduced risk of illness, while the employer will benefit from healthier, more productive workers and lower absenteeism. If proven cost-effective, this type of intervention may be implemented on a larger scale in the future. If proven not effective despite sufficient power to detect a clinically meaningful change in activity level, and subsequent health and productivity at work, the finding will help in allocating occupational health resources to be used otherwise.

## Competing interests

KR, AV and TA declare that they have no competing interests. ST is the founder and CEO in Evalua International Ltd. that was responsible for the health risk appraisal used and ST and WvM are directors of Evalua Nederland b.v.

## Authors' contributions

KR is the principal investigator, who developed the idea for the study, obtained funding for research and designed the conduct of the study. KR has authored the protocol and will be in charge of reporting the findings of this study. ST and AV conducted the power calculations and advised on statistical matters in designing the study. TA and WvM provided expert comments. ST provided advice and guidance on the study design, former research and the conduct of the study, and commented the study protocol. All authors have read and approved the final manuscript.

## APPENDIX 1

Exclusion criteria - physical testing

### Exclusion criteria

• Elevated resting blood pressure (BP)

◦ Systolic BP over 170 and diastolic BP over 110

◦ Systolic BP over 180 or diastolic BP over 110

• Fever at the time of testing

• Fever during the previous week

• Pregnancy

• Over 65 years of age

• Scheduled retirement in the next two years

• Subject has been recommended by the occupational physician not to take part in heavy exercise

### Subject fulfils at least 4 of the following risk factors

• First-degree relative has suffered a myocardial infarction, has undergone a coronary by-pass operation or angioplasty or died due to cardiovascular disease under the age of 55 years.

• current smoker (> 1 cigarette a day)

• serum cholesterol >5,2 (HDL cholesterol <0,9)

• resting blood pressure over 140/90

• diabetes

• morbid obesity (BMI>40, waist circumference >140 cm)

• sedentary lifestyle

### Study subject fulfils one of the following

• Beta blocker medication due to hypertension or arrhythmia. (Beta blocker treatment for migraine is not an exclusion criteria)

• Hypertension treated with at least 2 different drugs

• Coronary heart disease

• Cardiac valve disease

• Arrhythmia

• Pacemaker

• Heart failure

• Myocarditis

• Stroke

• Emphysema

• Chronic obstructive pulmonary disease

• Glycosylated haemoglobin A1c over 10

• Renal failure

• Chronic liver disease

• Claudication (Claudicatio intermittens)

• Cancer

### Study subject fulfils one of the following

• Myocardial infarction

• History of exercise-induced chest pain or shortness of breath without further examinations

• Disturbance in the cerebral blood flow within the past 12 months

• Major accidents or trauma within the last 6 months

• Major surgery within the last 6 months

### Study subject has one of the following medical problems

• Varfarin- treatment (Marevan) initiated within the past month or the treatment targets have not been reached

• Diabetes, diagnosed within the last month or the treatment targets have not been reached

• Increased ocular pressure (glaucoma)

• Rheumatoid arthritis in the active stage

• Unstable chronic liver disease

• Acute ventricular or duodenal ulcer

• Acute esophagitis

• Symptomatic anemia

• Acute hyperthyroiditis or other major thyroid problem

• Unstable asthma

## Pre-publication history

The pre-publication history for this paper can be accessed here:

http://www.biomedcentral.com/1471-2458/9/494/prepub
